# Identification of new DNA i-motif binding ligands through a fluorescent intercalator displacement assay†
†Electronic supplementary information (ESI) available: Experimental, supporting fluorescence and UV-Vis data, Job plot, binding curves, CD spectra, melting temperatures and SPR data. See DOI: 10.1039/c7ob00710h


**DOI:** 10.1039/c7ob00710h

**Published:** 2017-06-01

**Authors:** Qiran Sheng, Joseph C. Neaverson, Tasnim Mahmoud, Clare E. M. Stevenson, Susan E. Matthews, Zoë A. E. Waller

**Affiliations:** a School of Pharmacy , University of East Anglia , Norwich Research Park , Norwich , NR4 7TJ , UK . Email: z.waller@uea.ac.uk; b Department of Biological Chemistry , John Innes Centre , Norwich Research Park , Norwich , NR4 7UH , UK; c Centre for Molecular and Structural Biochemistry , University of East Anglia , Norwich Research Park , Norwich , NR4 7TJ , UK

## Abstract

i-Motifs are quadruplex DNA structures formed from sequences rich in cytosine and held together by intercalated, hemi-protonated cytosine–cytosine base pairs. These sequences are prevalent in gene promoter regions and may play a role in gene transcription. Targeting these structures with ligands could provide a novel way to target genetic disease but there are very few ligands which have been shown to interact with i-motif DNA. Fluorescent intercalator displacement (FID) assays are a simple way to screen ligands against DNA secondary structures. Here we characterise how thiazole orange interacts with i-motif DNA and assess its ability for use in a FID assay. Additionally, we report FID-based ligand screening using thiazole orange against the i-motif forming sequence from the human telomere to reveal new i-motif binding compounds which have the potential for further development.

## Abstract 2

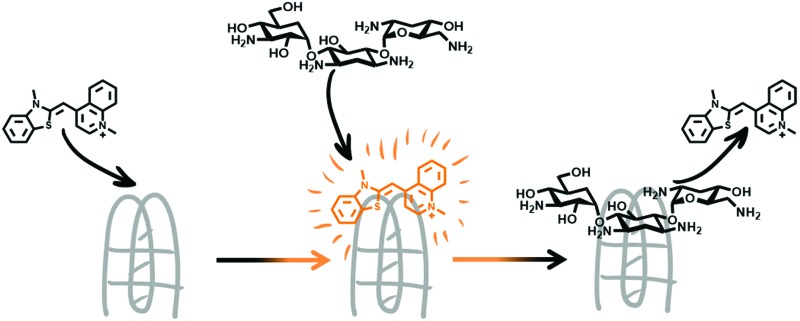
This work describes a new way to screen for i-motif binding compounds and several new families of ligands with potential for use in experiments into the structure and function of i-motif DNA.

## 

DNA sequences rich in cytosine are capable of forming i-motifs, non-canonical quadruplex secondary structures where two parallel stranded duplexes are held in an antiparallel manner by the intercalation of hemiprotonated cytosine–cytosine^+^ base pairs.^1^,^2^ i-Motif structures are pH-dependent, which has led to novel uses in nanotechnology.^3^–^5^ However, targeting the i-motif with ligands has not been well documented in the literature, mainly due to the acidic conditions usually required to stabilise the structure and the consequent conflicts associated with attempting to target a potentially biologically-relevant structure under physiological conditions.^2^ Nevertheless, there has been increasing evidence to suggest that i-motif structures can form at neutral and even slightly alkaline pH^6^ depending on the sequence,^7^ the presence of molecular crowding agents,^8^ conditions of negative superhelicity^9^ and different types of cations.^10^,^11^ Compounds which interact with i-motif have been shown to alter gene expression^12^ and alter telomerase activity,^13^ but studies like these are limited and restricted by the available choice of i-motif binding compounds.

Förster resonance energy transfer (FRET) based experiments have mainly been used as a method for identifying the effects ligands have on i-motif structure.^12^,^14^,^15^ Although these are well-established methods, the addition of fluorophores to the DNA is necessary and is not only more expensive than using unlabelled DNA, but the additional fluorophores can affect the folding and stability of the DNA secondary structure, thus potentially affecting the results obtained by these means.^16^ Fluorescent intercalator displacement (FID) assays have previously been developed to produce a low cost and high through-put method of screening compounds for their selectivity and affinity for DNA structures such as G-quadruplexes,^17^,^18^ triplexes,^19^ hairpins^20^ and double stranded DNA sequences.^21^ An FID assay relies on a non-covalent intercalator that fluoresces when bound to DNA but not when competitively displaced by a ligand. This loss of fluorescence can be detected by common microplate readers using 96- or 384-well plates, allowing screens to be conducted against a large number of putative ligands with multiple DNA structures and sequences. However, currently there are no reported examples of FID-type assays developed for i-motif structures. Here we disclose a FID-based method which can be used for screening for compounds which bind i-motif DNA.

In order to develop an FID assay for i-motif DNA it was first important to identify a suitable probe. Ideally, FID probes need to both bind the target of interest without affecting the structure and result in a significant fluorescence change on binding. To identify potential i-motif binding fluorescent probes we investigated previously used probes for DNA secondary structures:^18^ ethidium bromide,^19^,^21^ thiazole orange,^17^,^18^ acridine orange,^22^ crystal violet^23^ and a pyrene derivative^24^ (Fig. S1†). The potential probes were assessed for any changes in fluorescence on addition of an i-motif forming sequence of DNA from the human telomere (5′-d[TAA-CCC-TAA-CCC-TAA-CCC-TAA-CCC]-3′, hTeloC). Probes were examined in 10 mM sodium cacodylate buffer at pH 5.5, a pH at which most literature i-motif structures are stable.^2^

Of the probes examined, thiazole orange (TO) demonstrated the largest change in fluorescence on binding (Table S1†). In the absence of DNA, TO does not show any remarkable fluorescent properties but on titration of hTeloC at pH 5.5, a significant increase in fluorescence was observed (see Fig. 1). Further aliquots of hTeloC i-motif resulted in a steady increase in fluorescence emission, suggesting a concentration-depended binding event between TO and hTeloC. The experiment was also performed at pH 7.4, where hTeloC is not folded into i-motif conformation. An increase in fluorescence was observed, but the enhancement was not as great, indicative that binding to the folded structure of the i-motif is important for fluorescence enhancement. For the other probes examined, the fluorescent intensity did not display the enhancement observed with TO (Fig. S2†). As a result, TO was selected as the most suitable fluorescent probe and characterization of binding to the i-motif was performed. Further fluorescence experiments with TO and hTeloC across a wider pH range (5–8, Fig. S5†), indicated a pH of 5.5–6 for maximum fluorescence enhancement. This enhancement is likely due to the optimal formation of hTeloC i-motif structure at these pHs,^25^ consistent with binding of the folded structure.

**Fig. 1:**
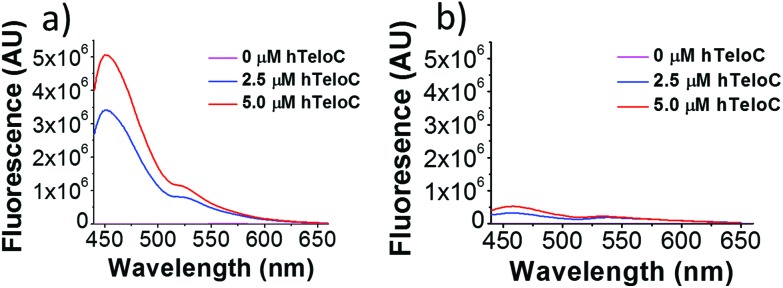
Fluorescence emission of TO (5 μM) in the presence of 0–5 μM hTeloC in 10 mM sodium cacodylate buffer at pH 5.5 (a) and pH 7.4 (b).

Using UV-Vis spectroscopy, the stoichiometry of binding between TO and hTeloC was determined using the method of continual variation binding analysis, indicating a 2 : 1 binding ratio of TO : hTeloC (see Fig. S7†). Binding affinity between TO and hTeloC was also determined using UV-Vis titrations. Starting with 5 μM of hTeloC in 50 mM sodium cacodylate at pH 5.5, small aliquots of TO were titrated in and the resulting spectrum taken. Using the change in absorbance at 505 nm, a hyperbolic binding curve of fraction bound *versus* TO concentration was generated. Given the known stoichiometry, the data was fitted with an independent two-site binding model to give two binding constants (*K*_d1_ = 3.7 ± 0.7 μM, *K*_d2_ = 78 ± 13 μM, Fig. S8†). These results reveal that TO binds with reduced affinity to i-motif compared to G-quadruplex and duplex DNA (which demonstrate *K*_d_s between 0.3 and 0.5 μM).^26^ Importantly, the initial fluorescence and binding studies indicated that TO could bind i-motif DNA strongly enough to observe good fluorescence, but weakly enough to be displaced by another ligand.

To investigate the ability of TO to alter or influence the conformation of i-motif DNA, circular dichroism (CD) was used. Experiments were performed at pH 5.5 (where most i-motif forming sequences are stable) the transitional pH (where 50% of the hTeloC is folded) and pH 7.4 (physiological pH). At and below the transitional pH (pH 6 and pH 5.5 Fig. S9 and S10†) intense positive signals are observed at 288 nm accompanied by negative signals at 258 nm, both characteristic of DNA folded into an i-motif conformation.^27^ At pH 5.5, titration of TO into hTeloC resulted in no significant changes in the signals, indicating the conformation remains constant on TO binding up to 50 μM. After this, a reduction in signals were observed, consistent with condensation of the ligand–DNA complex (Fig. S9†).^27^ A similar effect was observed at pH 6 (Fig. S10†). At pH 7.4 the signal at 288 nm is absent, indicating hTeloC is no longer folded into an i-motif (Fig. S11†). At this pH, the equilibrium is shifted towards a mixture of random coil and hairpin.^28^ On addition of TO to hTeloC at pH 7.4 a reduction in signal intensity appeared after the addition of 10 μM, after which a positive absorbance was also observed between 300 and 320 nm, indicative of a condensation event^27^ rather than any changes in structure. The results from the CD indicate that TO does not alter the conformation of hTeloC i-motif, so is suitable for use in an i-motif displacement based assay.

To investigate any stabilisation properties of TO, DNA melting experiments were conducted using FRET. In addition to the sequence from the human telomere 5′-FAM-d[TAA-CCC-TAA-CCC-TAA-CCC-TAA-CCC]-TAMRA-3′ (hTeloC_FRET_) we also examined two other previously described i-motif forming sequences: 5′-FAM-d[TCC-CCA-CCT-TCC-CCA-CCC-TCC-CCA-CCC-TCC-CCA]-TAMRA-3′ (c-MycC_FRET_), from the promoter region of MYC^9^,^15^ and 5′-FAM-d[CGC-GCT-CCC-GCC-CCC-TCT-CCC-CTC-CCC-GCG-C]-TAMRA-3′ (HIF-1αC_FRET_) from the promoter region of HIF-1α.^29^ Melting experiments were performed at the respective transitional pH and at pH 5.5. All experiments showed that TO has a stabilising effect on i-motif DNA, regardless of sequence or pH (see Fig. S10–12†); increasing the concentration of TO was found to increase the melting temperature. The stabilisation effect of adding TO was most profound in DNA sequences at the transitional pH (*i.e.* that were partially unfolded and inherently less stable) and thus assay buffer conditions below the transitional pH are preferred.

Initial fluorescent intercalator displacement assays were performed using 1 μM of pre-annealed hTeloC i-motif, combined with 2 μM of TO in 10 mM sodium cacodylate at pH 5.5. After an equilibration period of 5 minutes, the sample was titrated with candidate i-motif binding ligands and excited at 430 nm using a fluorimeter. Initial studies were performed using known i-motif binding ligand **mitoxantrone**.^15^ On addition of **mitoxantrone**, a significant loss in fluorescence emission at 450 nm was observed, indicative of displacement of TO from the DNA (Fig. 2a).

**Fig. 2:**
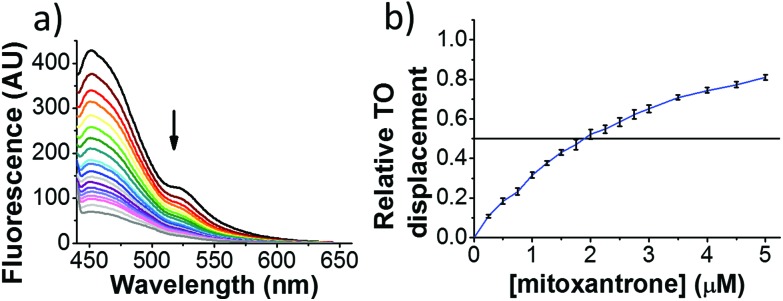
Example FID assay using hTeloC (1 μM), TO (2 μM) and **mitoxantrone** (0–5 μM) at pH 5.5 in 10 mM sodium cacodylate buffer. (a) Example raw fluorescence data. (b) Displacement *versus* concentration plot.

By varying the concentration of ligand added, a DC_50_, the concentration where 50 percent of the TO was displaced, can be calculated from a plot of *D*_*x*_ against the concentration of ligand. The results showed that the DC_50_ for **mitoxantrone** against hTeloC, was 1.8 μM, which is consistent with it being a moderate i-motif binding ligand.^15^

Next, a higher throughput screening experiment in a 384-well plate format using the TO probe and a 960 compound library. The MicroSource library used contains a wide range of known drugs, natural products and biologically active compounds, including **mitoxantrone**. In the screen, 0.5 μM (1 eq.) of pre-annealed hTeloC was mixed with 1.0 μM (2 eq.) of TO in the sodium cacodylate buffer in the wells. Then 2.5 μM (5 eq.) of the ligand from the compound library was added, mixed and scanned on a plate reader. TO displacement (*D*_TO_) was calculated for each compound and they were ranked according to their *D*_TO_. Example hits are shown in Table 1.

**Table 1:** Structures of the hit compounds from the FID assay and data for interaction with hTeloC in 10 mM sodium cacodylate at pH 5.5, % displacement of TO from the screening (*D*_TO_), DC_50_ and % binding (%*R*_max_) determined by SPR

Compound	*D*_TO_/%	DC_50_/μM	%*R*_max_ at 50 μM/%
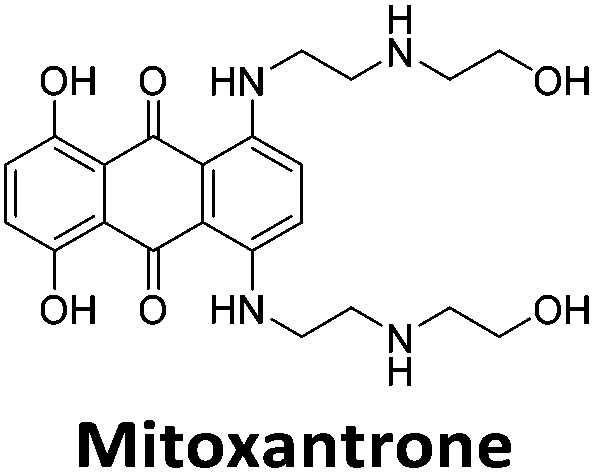	86	1.8	2125
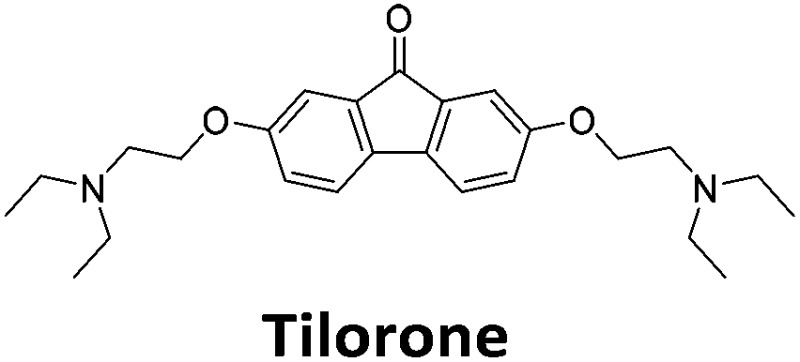	70	2.4	116
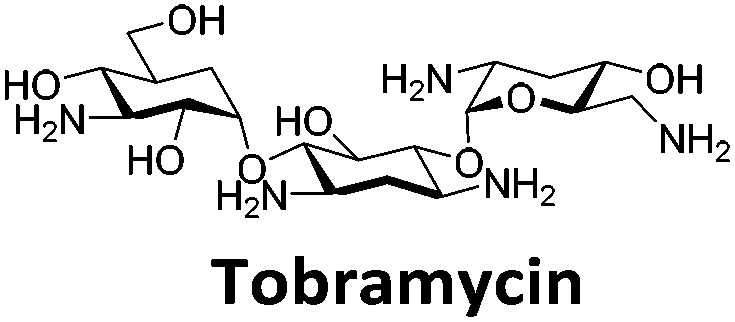	49	2.9	325
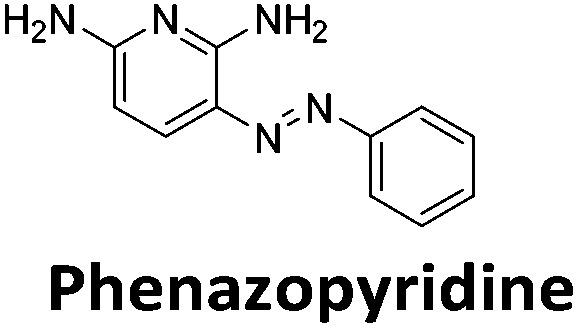	31	Nd	38
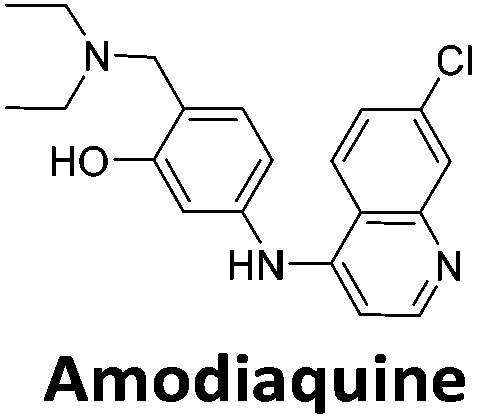	30	Nd	15
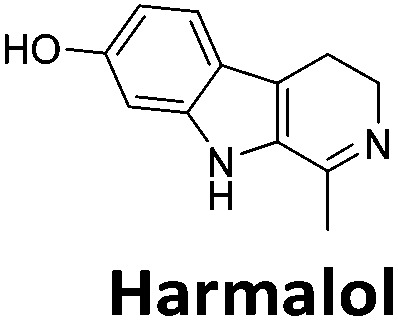	27	Nd	NB
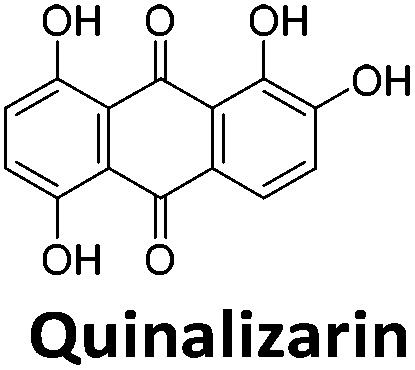	22	Nd	NB
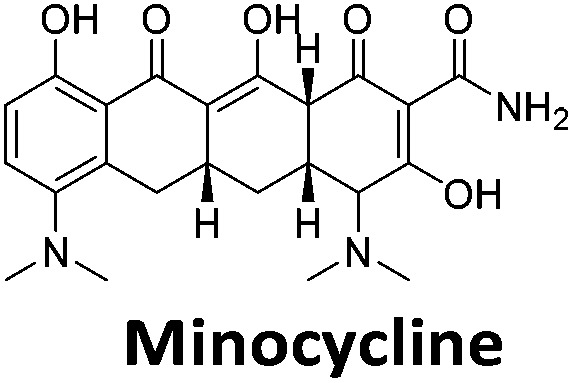	21	Nd	29
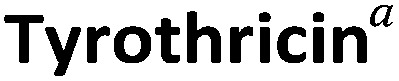	19	>5	7

^*a*^Tyrothricin is a mixture of cyclic peptide natural products from *Bacillus brevis*.

Similar to a previous FRET melting screen,^15^**mitoxantrone** was found to be one of the top hits in the FID screen with TO displacement of 86% This was followed by two bisbiguanides: **chlorhexidine** and **alexidine** which are known surfactants^30^,^31^ and likely to be condensing the DNA in an unspecific fashion (see ESI†). The next-best ligand in this library after **mitoxantrone** was found to be **tilorone**, with a *D*_TO_ of 70% followed by **tobramycin** with a *D*_TO_ of just under 50% The remaining ligands gave a *D*_TO_ of less than 50%, indicating weaker binding compounds which are less able to compete with TO for the i-motif binding sites. This indicates that TO is able to act as a threshold to eliminate weak i-motif binders. Full FID displacement titrations were performed for **tilorone** and **tobramycin** which gave a DC_50_ of 2.4 μM and 2.9 μM respectively. A much weaker ligand, **tyrothricin**, was also measured over a concentration range, but there was no significant displacement up to 5 μM, consistent with the FID screen. In the FID assay, when tested ligands are added to the solution of TO, there are several potential reasons for the loss of fluorescence including: effective displacement of TO, ligand-induced condensation or precipitation and a strong absorption at approximately 450 nm. Determining the UV-Vis absorbance of any “hit” compounds can be easily measured to check for false positives *via* this mechanism. We examined all the hits for their UV-Vis properties and **phenazopyridine** (31% displacement), was found to also have an absorption in the same region as the TO excitation wavelength, indicating a possible false positive result. Ligand binding which induces condensation cannot be discriminated from direct displacement in any FID assay. Likewise, if a ligand interferes with the fluorescence of TO, this can also mislead readings. These are inherent drawback of the technique and any hits should also be assessed using a different method. Nevertheless, this assay provides a quick and inexpensive preliminary screening method for identification of potential i-motif binding ligands.

To give an indication of how these results compare with another method to determine binding, surface plasmon resonance (SPR) was undertaken. SPR experiments were performed using three different immobilised DNA targets: hTeloC_Biotin_ (5′-biotin-[TAA-CCC-TAA-CCC-TAA-CCC-TAA-CCC]-3′), c-MycC_Biotin_ (5′-biotin-[CCT-TCC-CCA-CCC-TCC-CCA-CCC-TCC-CCA]-3′) and also double stranded DNA (DS_biotin_) for comparison, which comprised the ODN d(biotin-[GGC-ATA-GTG-CGT-GGG-CGT-TAG-C]) hybridized with its complementary strand. To compare across a number of ligands of different affinities, a single concentration (50 μM) was used to assess binding against i-motif forming sequences. The responses were recorded at equilibrium and compared to the predicted binding response (*R*_max_) calculated with a 1 : 1 binding stoichiometry. The results for binding to hTeloC_biotin_ are given in Table 1 (full Table in ESI†). The percentage of predicted maximum response (%*R*_max_) is an indicator for binding affinity. Of all the compounds tested, **mitoxantrone** has the highest %*R*_max_ (2125%), followed by **tobramycin** (325%) and **tilorone** (116%). **Phenazopyridine**, **amodiaquine**, **minocycline** and **tyrothricin** also showed evidence of binding at 50 μM. **Harmalol** and **quinalizarin** did not show significant binding at this concentration with hTeloC_biotin_, but did with cMycC_biotin_ (Table S2†). The SPR relative binding results are consistent with the trends found using the FID screen, providing some validation to the procedure.

Additional SPR experiments were performed to determine the affinity of binding of **tobramycin** (Fig. S16†) and **tilorone** (Fig. S15†). **Tobramycin** was found to bind hTeloC_biotin_ with a *K*_d_ of 17 ± 2.0 μM, which was with similar affinity as c-mycC_biotin_ with a *K*_d_ of 13 ± 1.8 μM. Given the nature of the compound library which houses known drugs which affect traditional drug targets, it is unsurprising that **tobramycin** was also found to bind double stranded DNA with a *K*_d_ of 18 ± 1.1 μM. Further assessment of **tilorone** by SPR was also performed, but it was found to be significantly weaker and because of this could not be accurately determined using the same range of concentrations (Fig. S17†). The screen indicates the suitability of TO as a probe for i-motif binding and has identified several novel i-motif binding ligands. Given the scant literature surrounding compounds which interact with i-motif, these newly identified ligands offer much promise as leads for further development to target i-motif DNA.

## Conclusions

Herein we have described the characterization of TO binding against i-motif DNA. The compound was found to bind with a 2 : 1 stoichiometry with low micromolar dissociation constant, making it suitable for use in a FID displacement assay. An example screen using a library of known drugs, natural products and biologically active compounds identified several new i-motif binding ligands which have potential as lead compounds to develop in the use in the study of i-motif DNA structure and function.

## Supplementary Material

Supplementary information
