# Supplementary material for: Identification of new DNA i-motif binding ligands through a fluorescent intercalator displacement assay
Source: Org Biomol Chem. 2017 Jun 1;15(27):5669–73. doi: 10.1039/c7ob00710h (PMC5708337; doi:10.1039/c7ob00710h)
Supplement: Supplementary file 1 [file OB-015-C7OB00710H-s001.pdf]

Electronic Supplementary Material (ESI) for Organic & Biomolecular Chemistry.
This journal is © The Royal Society of Chemistry 2017

SUPPORTING INFORMATION

Identification of new DNA i-motif binding ligands through a fluorescent intercalator displacement assay

Qiran Sheng,† Joseph C. Neaverson,† Tasnim Mahmoud,† Clare E. M. Stevenson,¥ Susan E. Matthews,† and Zoë A. E. Waller*†§

†School of Pharmacy, University of East Anglia, Norwich Research Park, Norwich, NR4 7TJ, UK

¥Department of Biological Chemistry, John Innes Centre, Norwich Research Park, Norwich, NR4 7UH, UK

§Centre for Molecular and Structural Biochemistry, University of East Anglia, Norwich Research Park, Norwich, NR4 7TJ, UK

EXPERIMENTAL PROCEDURES

1) GENERAL EXPERIMENTAL

All the oligonucleotides (ODNs) and their fluorescent conjugates were purchased from Eurogentec and were HPLC purified. Solid DNA samples were initially dissolved as a stock solution in MilliQ water (100 µM for labelled and 1 mM for un-labelled ODNs); further dilutions were carried out in 10 mM sodium cacodylate buffer at the respective pH. Annealed samples were thermally annealed in a heat block at 95°C for 5 minutes and cooled slowly to room temperature overnight. Candidate FID probes **acridine orange**, **thiazole orange** and **crystal violet** were purchased from Sigma Aldrich. **Ethidum bromide** and **1-pyrenemethanol** were purchased from Fisher Scientific. Stock solutions of ligands at 10 mM were made in DMSO and were stored at -20°C, subsequent dilutions were made in the appropriate buffer. The Gen-Plus library from Microsource Discovery Systems Inc. consisting of 960 drug standards with approval in Europe, Japan or the USA was supplied as 10 mM solutions in DMSO which were diluted to 1 mM in 96 well plates. Data manipulation was performed using Origin 8.0.

2) FLUORESCENCE SPECTROSCOPY

Fluorescence spectra were measured on a Horiba Jobin Yvon Fluorolog a Perkin-Elmer LS-55 fluorescence spectrometer with a Starna Scientific type 28/9-F 1 cm path length quartz cuvette. All experiments were performed in triplicate. DMSO stock solutions of the probes were diluted to 2.5 µM in buffer at the desired pH. hTeloC (5'-d[TAA-CCC-TAA-CCC-TAA-CCC-TAA-CCC]-3') DNA samples were prepared at 500 µM in buffer at the respective pH. In the experiments, 200 µL of probe (2.5 µM in buffer) was aliquoted into a cuvette and excitation and emission spectra of the ligand-DNA complex were measured. Then, 1 µL of the prepared solution of DNA (2.5 µM) was added to mimic the 1:1 binding event. After mixing and allowing 5 minutes to equilibrate, excitation and emission spectra of the ligand-DNA complex were measured again.

Fluorescence enhancement measurements using TO were performed using TO diluted in the respective buffer. DNA samples of hTeloC and c-MYCC (5'-d[TCC-CCA-CCT-TCC-CCA-CCC-TCC-CCA-CCC-TCC-CCA]-3') were diluted to 100 µM in buffer at the respective pH and annealed. In the experiments, 200 µL of TO (5 or 2.5 µM) was added to the cuvette and an emission spectrum was taken to observe the fluorescence in the buffer conditions in the absence of DNA. DNA was titrated into the sample, mixed, allowed to equilibrate for 5 minutes after each addition, then excited at 430 nm; fluorescence emission spectra were measured from 440 to 650 nm.

3) UV-VIS SPECTROSCOPY

Samples were measured using on an Agilent Technologies Cary 60 UV-Vis spectrophotometer with a Starna Scientific 1 cm path length quartz cuvette over a range of 350-550 nm at a rate of 300 nm/s with a data interval of 0.5 nm with a dual beam at room temperature. Samples containing only buffer were measured and subtracted from the data.

Stoichiometry was determined using the method of continuous variation binding analysis. 200 µL samples containing hTeloC DNA with TO were made up in pH 5.5 buffer. The individual concentrations of DNA and TO varied between 0 and 20 µM but the total concentration of DNA + TO remained constant at 20 µM. Job plots were constructed by plotting absorbance at 497.5 nm against the ratio of TO to hTeloC i.e. [TO]/([TO] + [hTeloC]).

UV-Vis titrations to determine binding affinity were performed using hTeloC (5 µM) in buffer at pH 5.5. A concentrated stock of TO prepared in buffer at pH 5.5 was prepared for titration into 200 µL of hTeloC to give final TO concentrations of 0, 1.25, 5.2, 3.75, 5, 7.5, 10, 15 and 20 µM. An analogous titration without DNA was subtracted from the data. Binding data were fitted using non-linear regression.

4) CIRCULAR DICHROISM SPECTROSCOPY

hTeloC (10 µM) was diluted in buffer at pH 5.5, pH 6.0 and pH 7.4 to give a total volume of 200 µL for each sample and were annealed overnight. Spectra were recorded using a Jasco J-810 spectropolorimeter with a Starna Scientific type-21 quartz cuvette of 1 mm path length. Scans were performed at 20°C from 220 nm to 320 nm with a scanning speed of 200 nm/min, a response time of 1 s, pitch of 0.5 nm and a bandwidth of 2 nm. Blank samples containing only the respective buffer were recorded and subtracted from the data. For each sample, TO or tobramycin was titrated directly into the cuvette containing the DNA and solutions were thoroughly mixed before recording spectra. All spectra show an average of three scans.

5) FRET MELTING EXPERIMENTS

The labelled oligonucleotides $hTeloC_{FRET}$ (5′-FAM-d[TAA-CCC-TAA-CCC-TAA-CCC-TAA-CCC]-TAMRA-3′, $c\text{-}Myc_{FRET}$ (5′-FAM-d[TCC-CCA-CCT-TCC-CCA-CCC-TCC-CCA-CCC-TCC-CCA]-TAMRA-3′), $hTeloG_{FRET}$ (5′-FAM-d[GGG-TTA-GGG-TTA-GGG-TTA-GGG]-TAMRA-3′) and DS_{FRET} FAM-d(TAT-AGC-TAT-A-HEG(18)-TAT-AGC-TAT-A)-TAMRA-3′); donor fluorophore FAM is 6-carboxyfluorescein; acceptor fluorophore TAMRA is 6-carboxytetramethyl-rhodamine; were prepared as a 400 nM solution in buffer at the respective pH and then annealed. Strip-tubes (QIAgen) were prepared by aliquoting 10 µL of the annealed DNA, followed by 10 µL of TO solutions made in the same buffer. Control samples for each

run were prepared without TO. Fluorescence melting curves were determined in a QIAgen Rotor-Gene Q-series PCR machine, using a total reaction volume of 20 µL. Samples were held at 25°C for 5 minutes then ramped to 95°C at increments of 1°C, holding the temperature at each step for 1 minute. Measurements were made with excitation at 470 nm and detection at 510 nm. DNA melting points were determined using the first derivative of the melting curve, any experiments where the inflection point was not able to be determined (i.e. the transition does not occur before the end of the experiment) were defined to have a T_m of >95°C. Final analysis of the data was carried out using QIAgen Rotor-Gene Q-series software and Origin or Excel.

6) FID ASSAY

TO was diluted to 2 µM in buffer at pH 5.5 and hTeloC i-motif was diluted to 50 µM. The tested ligands were diluted to 50 µM in buffer at pH 5.5. 196 µL of the TO solution was excited at 430 nm and the background fluorescence recorded from 450 to 650 nm. The background fluorescence emission intensity at 450 nm was normalised as 0%. Then 4 µL of hTeloC was added, mixed, allowed to equilibrate for 5 minutes and a second background fluorescence spectrum was taken when the sample was excited at 430 nm. The fluorescence emission intensity at 450 nm was normalized as 100% fluorescence. Then 1 µL aliquots of ligand were titrated into the sample and a spectrum measured. TO displacement was calculated using Equation S1 and plotted against concentration to calculate the DC_{50}.

Equation S1

$$D_x = 1 - \frac{F_x}{F_0} = 1 - \frac{F_{read} - F_{c_0}}{F_{refereence} - F_{c_0}}$$

The IM-FID assay was conducted using 384-well microplates (Corning® Low Volume 384 well Black Flat Bottom Polystryrene NBS TM Microplate) at 25°C. Microplate wells were filled with 40 µL of a testing solution consisting of hTeloC (0.5 µM) and TO (1.0 µM) in buffer at pH 5.5. Then 0.5 µL of library compound solution (1 mM in DMSO) was added into each well. Each plate had three control wells of DNA in buffer and another three reference wells of DNA and TO in buffer without ligand. After mixing, plates were read on a BMG CLARIOstar using an excitation filter from 400 to 430 nm and an emission filter from 460 to 480 nm. Each scan was performed three times. The basal fluorescence signal (F_{c0}) were assigned as the average fluorescence intensity read from the control wells. The 100% fluorescence intensity read ($F_{reference}$) was assigned as the average fluorescence intensity read from reference wells. The DC_{50} for each compound was calculated using the average of three reads (F_{read}) using Equation 1. Hit compounds were ranked according to DC_{50} (Supporting information).

7) SURFACE PLASMON RESONANCE

SPR experiments were performed using a GE Healthcare Biacore T200 instrument with a series S streptavidin (SA) coated chip. For immobilization all DNA samples were biotinylated. hTeloC$_{Biotin}$, (5′-biotin-d[TAA-CCC-TAA-CCC-TAA-CCC-TAA-CCC]-3′) c-Myc$_{Biotin}$ (5′-biotin-d[CCT-TCC-CCA-CCC-TCC-CCA-CCC-TCC-CCA]-3′) sequences were diluted to 1 µM in running buffer (10 mM sodium cacodylate (pH 5.5), 100 mM NaCl and 0.05% Tween-20) and the double stranded DNA DS$_{Biotin}$ (5′-biotin-d[GGC-ATA-GTG-CGT-GGG-CGT-TAG-C]-3′) was annealed with its complimentary strand at 1 µM in running buffer. For immobilization, the chip was first conditioned with three 60 s washes of 1 M NaCl and 50 mM NaOH at a flow rate of 10 µL min^{-1} to remove any unconjugated streptavidin. The biotinylated oligonucleotides were then injected over flow cells 2 (hTeloC$_{Biotin}$, 761.7 RU), 3 (c-Myc$_{Biotin}$, 664.0 RU) and 4 (DS$_{Biotin}$+comp, 568.1 RU) with flow cell 1 left blank.

For ligand screening, hit compounds were prepared in running buffer (10 mM sodium cacodylate (pH 5.5), 100 mM NaCl, 0.05% Tween-20 and 5% DMSO) and tested at 50 µM. The theoretical R_{max} was calculated using equation S2

$$R_{max} = \frac{MW_{compound}}{MW_{oligo}} \times RU_{oligo} \times i$$

Equation S2 .

Where $MW_{compound}$ = molecular weight of tested compounds, MW_{oligo} = molecular weight of the immobilised oligonucleotides on the chip. RU_{oligo} is immobilization level of the oligo on the chip in resonance units and *i* is the stoichiometry of binding (assumed to be 1:1 for the purposes of the screen).

For affinity measurements, the running buffer was identical. Ligand stocks (10 mM in DMSO) were diluted without DMSO to give a stock of each compound at 0.5 mM in running buffer. Serial dilution of this was then carried out with running buffer (10 mM sodium cacodylate (pH 5.5), 100 mM NaCl, 0.05% Tween-20 and 5% DMSO) to prepare concentrations of 200, 100, 50, 25, 12.5, 6.25, 3.125, 1.56, 0.78, 0.39 and 0 µM in a final composition the same as the running buffer. It was crucial that all concentrations of ligand contained 5% DMSO and in addition solvent correction was performed where 8 solutions with varying amounts of DMSO (4.5-5.8%) were also prepared. The solvent correction samples were run at the start and end of the experiment and every 30 cycles. Binding experiments were performed using the affinity run wizard in the Biacore T200 software at 25°C and a flow rate of 30 µL min^{-1}. Prior to sample injection, 1 startup cycle was performed: blank injections of buffer followed by 2 regeneration injections of 1 M NaCl. Each concentration of ligand was

injected for 120 s and the responses in each flow cell were measured. After each injection the chip surface was regenerated by a 60 s injection of 1 M NaCl followed by washing with running buffer for 60 s.. The response data was solvent corrected and double referenced by subtracting the startup cycle and injections of buffer only samples. Non-selective binding to the chip surface was accounted for by subtracting the response from the blank flow cell. Resultant sensorgrams were fitted using the average equilibrium response for each concentration and fitted using the affinity fit from the Biacore T200 evaluation software v2.0 assuming a 1:1 binding model.

SUPPLEMENTARY DATA

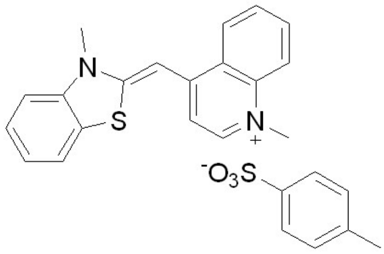

thiazole orange

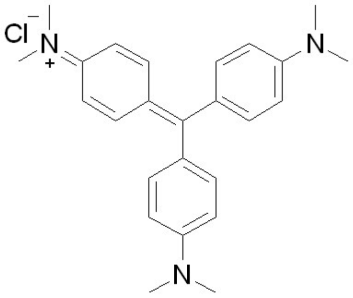

crystal violet

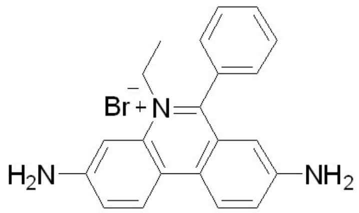

ethidium bromide

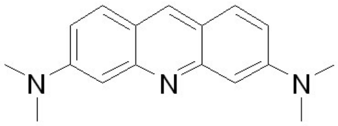

acridine orange

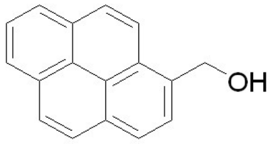

1-pyrenemethanol

Figure S1: Structures of probes examined using fluorescence

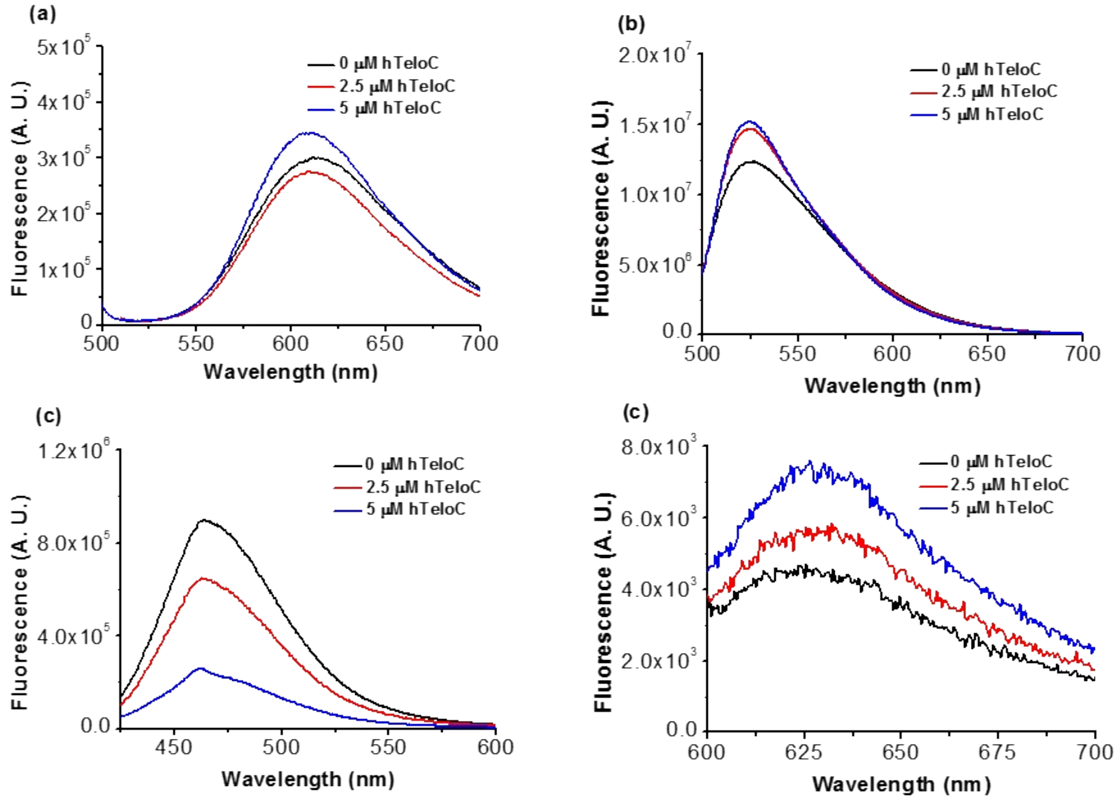

Figure S2: Example emission spectra of probes (0 - 5 μM) and hTeloC (5 μM) in 10 mM sodium cacodylate buffer, pH 5.5. a) ethidium bromide (λ_{ex} 490 nm); b) acridine orange (λ_{ex} 490 nm); c) 1-pyrenemethanol (λ_{ex} 440 nm); d) crystal violet (λ_{ex} 580 nm).

Compound	Excitation λ (nm)	Emission λ (nm)	Fluorescent Emission enhancement (%)
Acridine orange	490	520	23
Crystal violet	580	625	70
Ethidum bromide	490	620	-12
Thiazole orange	430	450	11860
1-pyrenemethanol	440	470	-74

Table S1: Fluorescence emission enhancement upon probe (5 µM) binding to hTeloC (5 µM) in 10 mM sodium cacodylate pH 5.5.

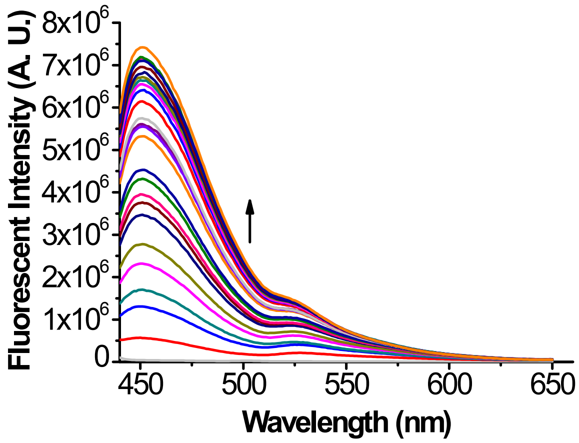

Figure S3: Fluorescence emission spectra of thiazole orange (5 µM) titrated with hTeloC (0 - 20 µM) at pH 5.5 in 10 mM sodium cacodylate buffer. Samples were excited at 430 nm.

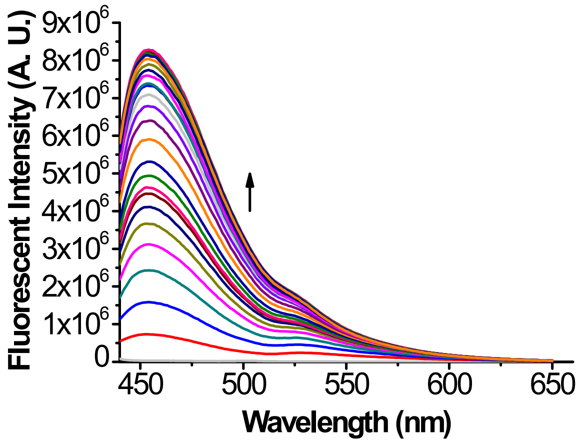

Figure S4: Fluorescence emission spectra of thiazole orange (5 µM) titrated with c-MYCC (0 - 20 µM) at pH 5.5 in 10 mM sodium cacodylate buffer. Samples were excited at 430 nm.

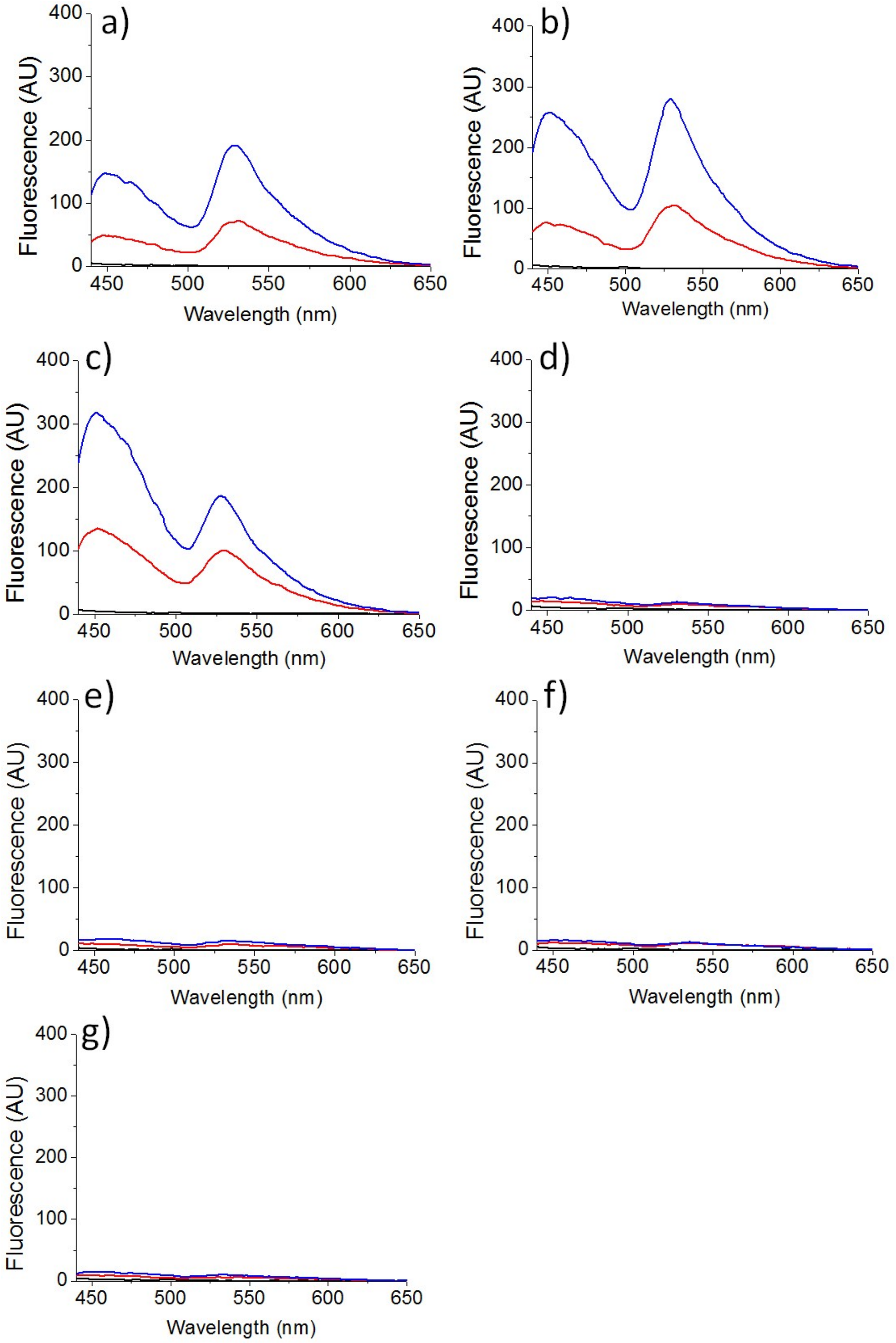

Figure S5: Example fluorescence emission spectra of thiazole orange (2.5 µM) with 0 (black), 1.25 (red) and 2.5 (blue) µM of hTeloC in 10 mM sodium cacodylate buffer at pH 5.0 (a), 5.5 (b), 6.0 (c), 6.5 (d), 7.0 (e), 7.5 (f) or 8 (g). Samples were excited at 430 nm.

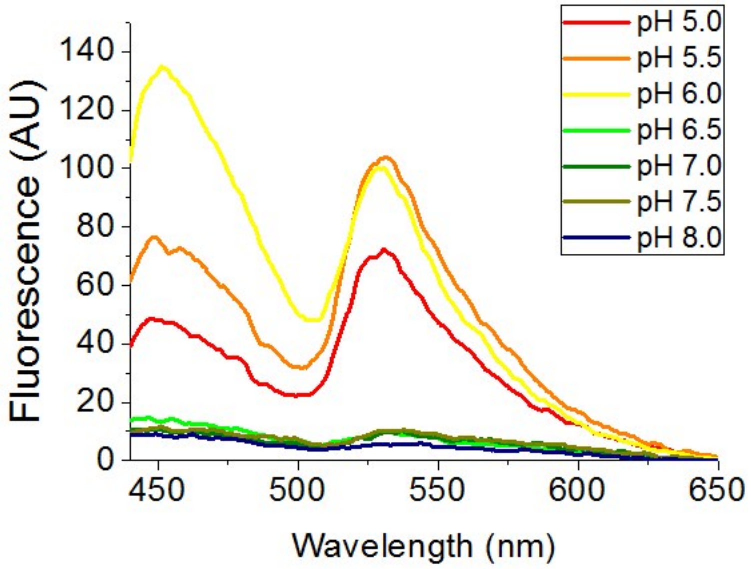

Figure S6: Example fluorescence emission spectra of thiazole orange (2.5 µM) with 1.25 µM of hTeloC in 10 mM sodium cacodylate buffer at pH 5-8. Samples were excited at 430 nm.

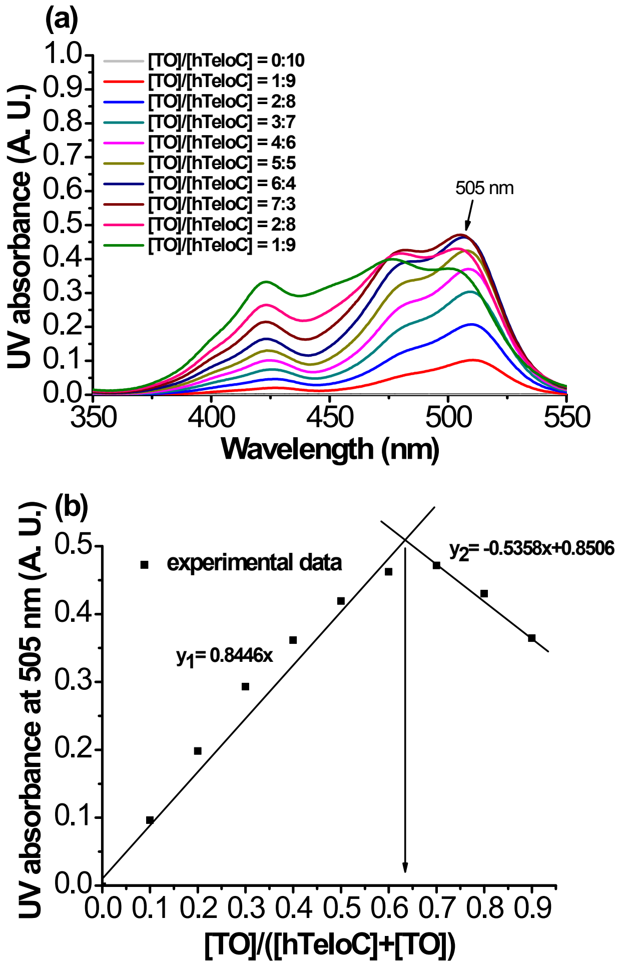

Figure S7: a) Example UV-Vis spectra of hTeloC and thiazole orange at pH 5.5 in 50 mM sodium cacodylate buffer; b) "Job Plot" for thiazole orange-hTeloC titration, indicating 2:1 stoichiometry

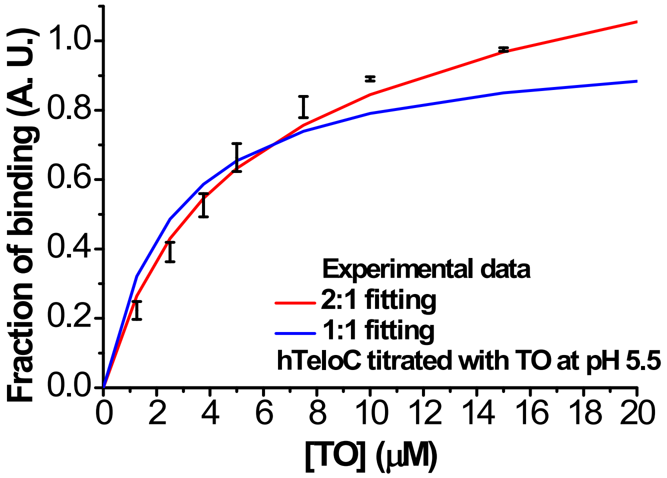

Figure S8: Binding data from the UV-Vis titration of thiazole orange with hTeloC (10 μM) at pH 5.5 in 50 mM sodium cacodylate buffer. 1:1 fitting was using Equation S3, 2:1 fitting was using Equation S4.

Equation S3

$$\theta = \frac{nK_1[Ligand]}{1 + K_1[Ligand]}$$

Where: θ = the fraction of binding measured; K_1 = the equilibrium association constant for the binding site; n = the stoichiometry.

Equation S4

$$\theta = \frac{K_1[Ligand] + 2K_1K_2[Ligand}{1 + K_1[Ligand] + K_1K_2[Ligan}$$

Where: θ = the fraction of binding measured; K_1 = the equilibrium association constant for the first binding site; K_2 = the equilibrium association constant for the second binding site.

The results from the binding studies support the model that two thiazole orange molecules bind to one i-motif.

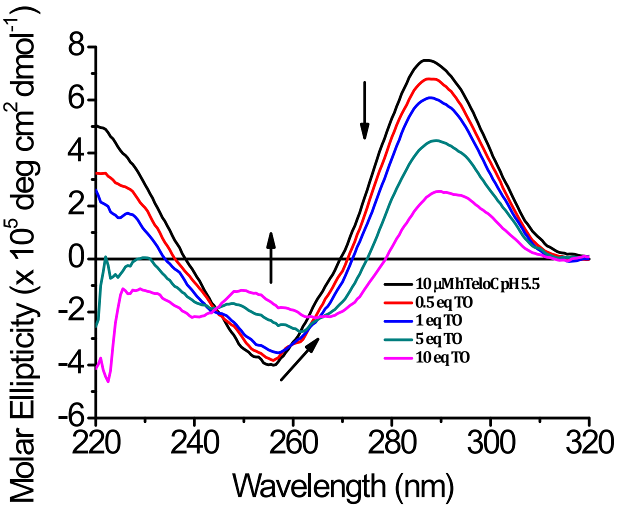

Figure S9: Circular dichroism titration of hTeloC (10 μM) with thiazole orange (0 – 100 μM) in 10 mM sodium cacodylate buffer pH 5.5.

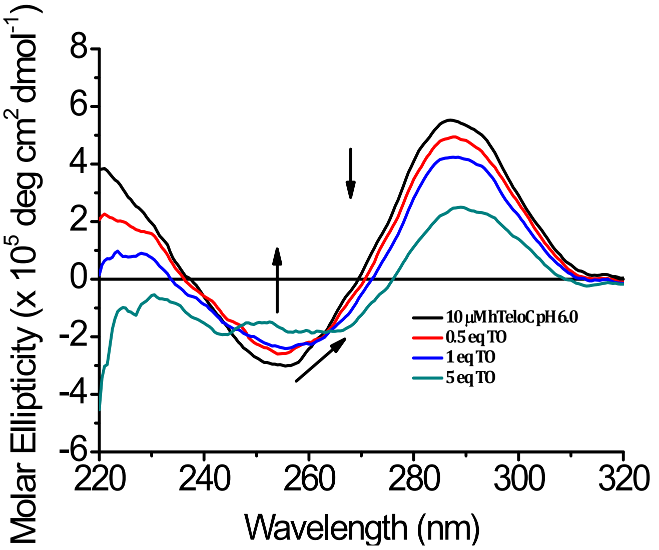

Figure S10: Circular dichroism titration of hTeloC (10 μM) with thiazole orange (0 – 50 μM) in 10 mM sodium cacodylate buffer pH 6.0.

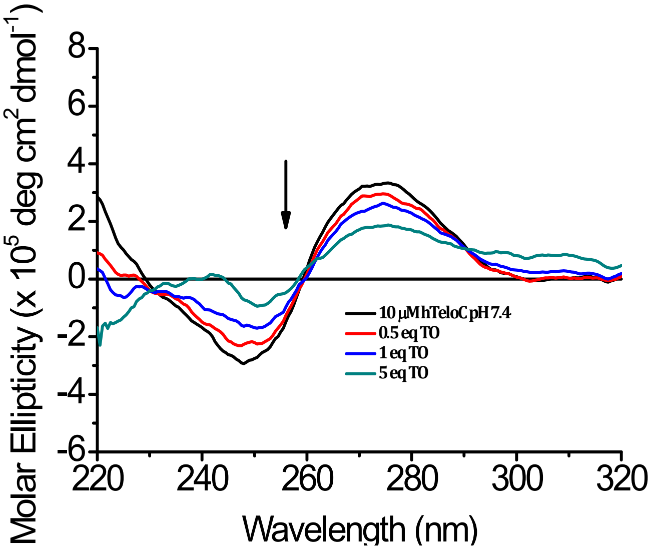

Figure S11: Circular dichroism titration of hTeloC (10 µM) with thiazole orange (0 - 50 µM) in 10mM sodium cacodylate buffer pH 7.4.

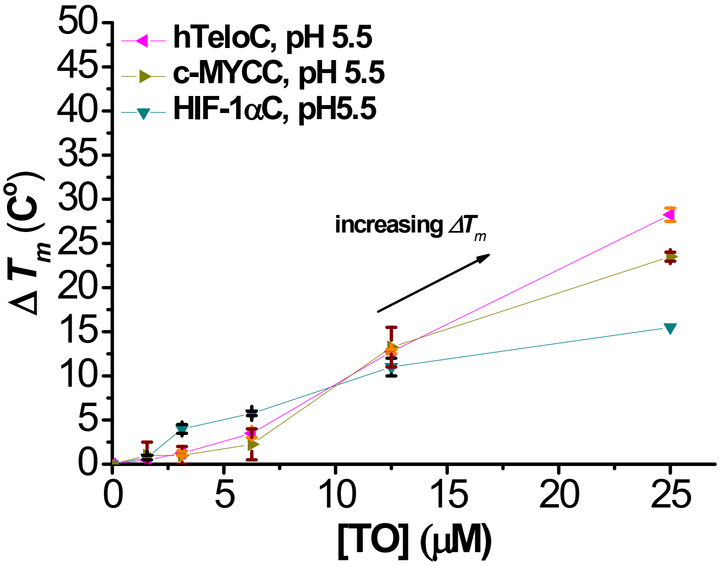

Figure S12: ΔT_m vs concentration plots determined by FRET-based DNA melting for hTeloC, c-MYCC and HIF-1-αC (200 nM) with TO in 50 mM sodium cacodylate buffer at pH 5.5.

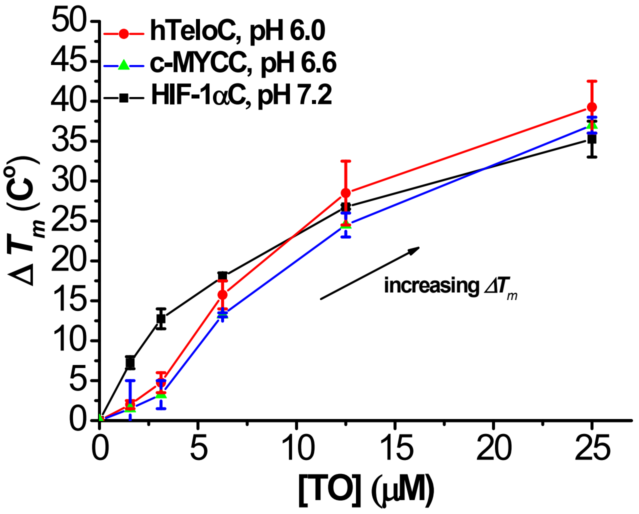

Figure S13: ΔT_m vs concentration plots determined by FRET-based DNA melting for hTeloC, c-MYCC and HIF-1-αC (200 nM) with TO in 50 mM sodium cacodylate buffer at transitional pH.

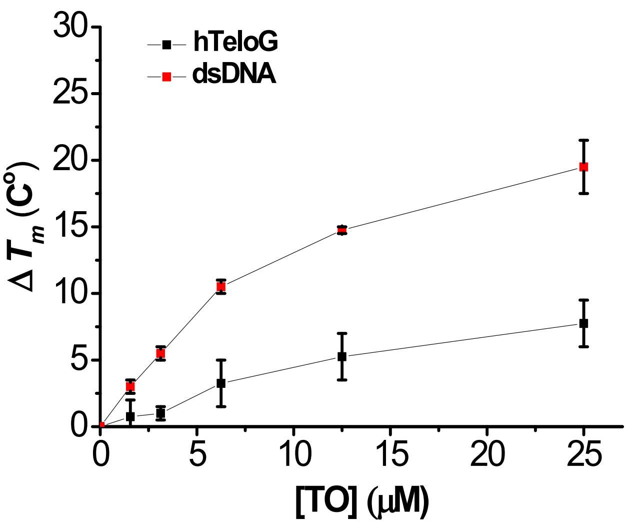

Figure S14: ΔT_m vs concentration plots determined by FRET-based DNA melting for hTeloG and dsDNA (200 nM) with TO in 50 mM sodium cacodylate buffer at pH 7.4.

Table S2: Structures of the hit compounds from the FID assay and data for interaction with hTeloC in 10 mM sodium cacodylate at pH 5.5, % displacement of TO from the screening (D_{TO}), DC_{50} and % binding (%R_{max}) determined by SPR in pH 5.5 10 mM sodium cacodylate, 100 mM NaCl, 0.05% Tween-20 and 5% DMSO.

Compound	D_{TO} /%	DC_{50} /µM	%R_{max} at 50 µM /%		
			hTeloC	c-MycC	dsDNA
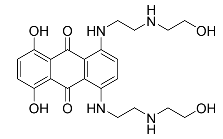 **Mitoxantrone**	86	1.8	2125	2534	4894
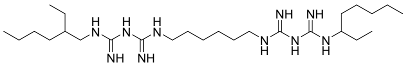 **Alexidine**	83	nd	nd	nd	nd
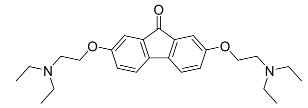 **Tilorone**	70	2.4	116	420	811
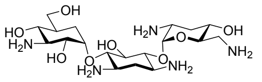 **Tobramycin**	49	2.9	325	121	233
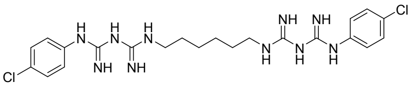 **Chlorhexidine**	42	nd	nd	nd	nd
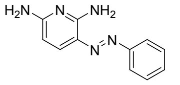 **Phenazopyridine**	31	nd	38	71	137
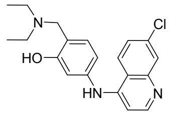 **Amodiaquine**	30	nd	15	31	59
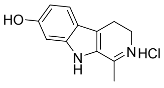 **Harmalol**	27	nd	NB	64	123
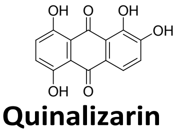 **Quinalizarin**	22	nd	NB	49	95
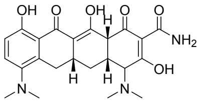 **Minocycline**	21	nd	29	36	69
**Tyrothricin**	19	>5	7	14	27

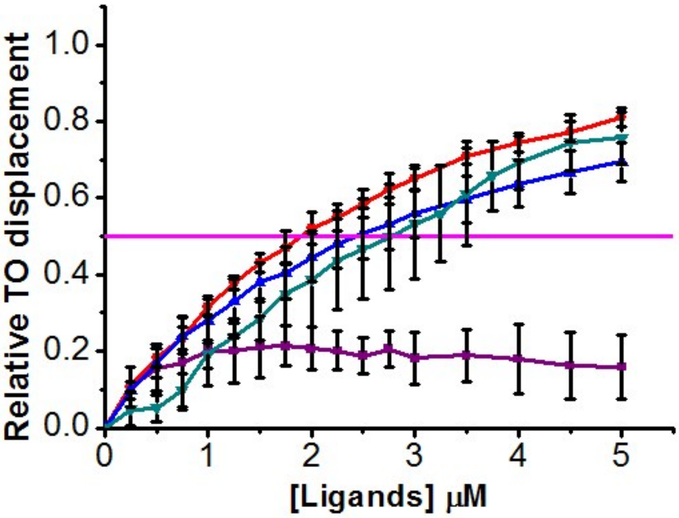

Figure S15: TO displacement versus concentration plot for mitoxantrone (red), tilorone (blue), tobramycin (green) and tyrothricin (purple) using hTeloC (1 μM) TO (2 μM) and ligand (0-5 μM) at pH 5.5 in 10 mM sodium cacodylate buffer.

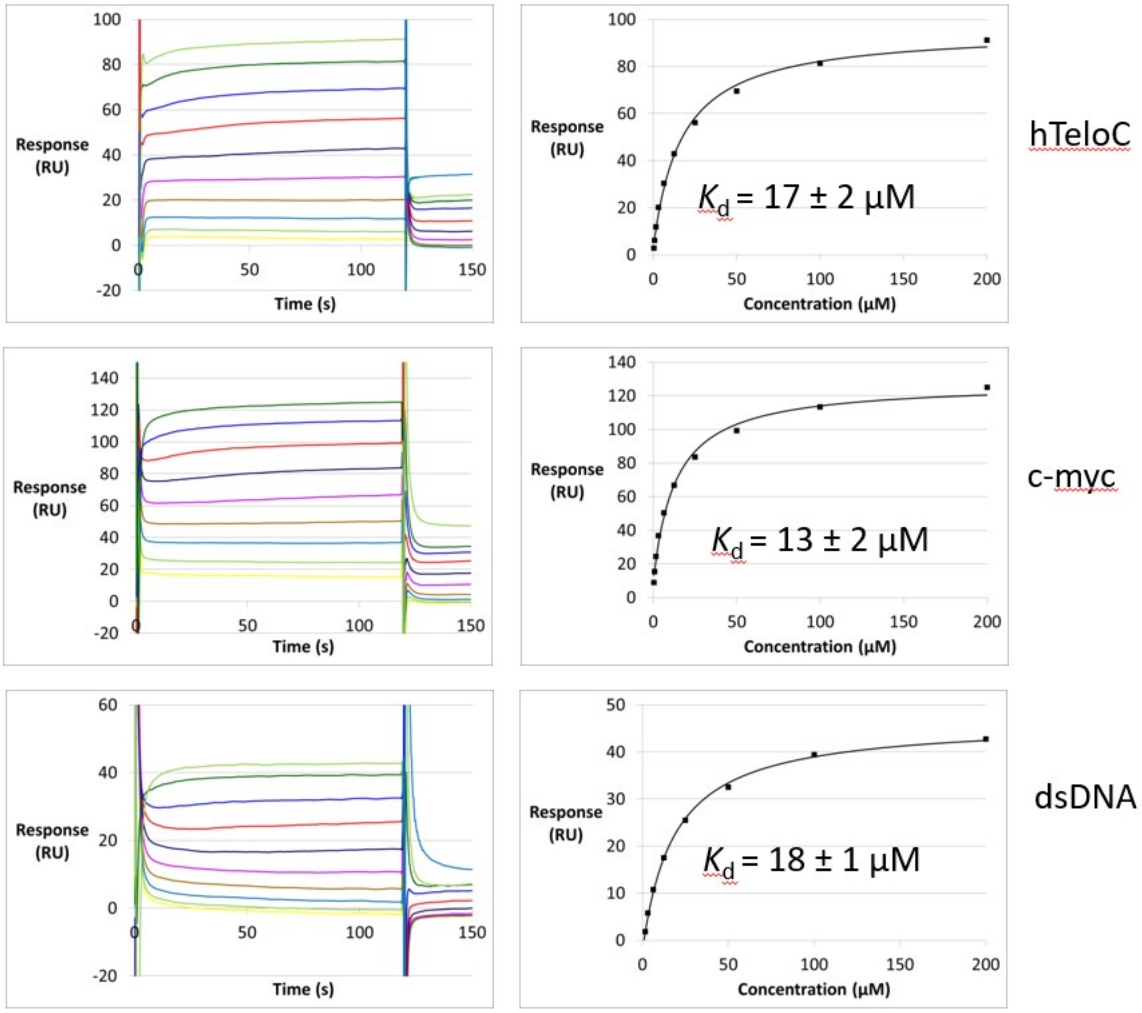

Figure S16: Example sensorgrams (left) and fittings (right) for tobramycin with hTeloC$_{biotin}$ (top) c-myc$_{biotin}$ (middle) and DS$_{biotin}$ (bottom) in pH 5.5 10 mM sodium cacodylate supplemented with 100 mM NaCl, 0.05% tween-20 and 5% DMSO. Sensorgrams are double referenced and solvent corrected.

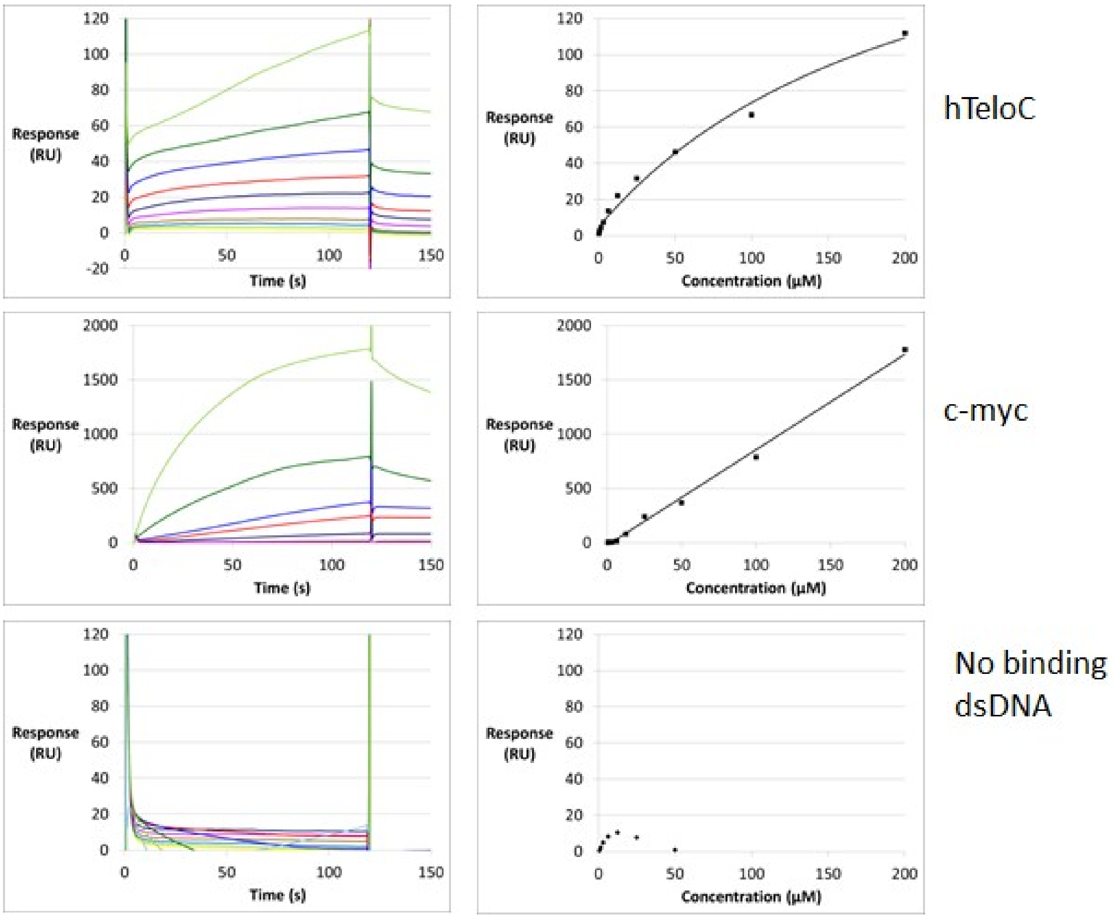

Figure S17: Example sensorgrams (left) and fittings (right) for tilorone with hTeloC$_{biotin}$ (top) c-myc$_{biotin}$ (middle) and DS$_{biotin}$ (bottom) in pH 5.5 10 mM sodium cacodylate supplemented with 100 mM NaCl, 0.05% tween-20 and 5% DMSO. Sensorgrams are double referenced and solvent corrected.
